# Gearbox Fault Diagnosis Based on Hierarchical Instantaneous Energy Density Dispersion Entropy and Dynamic Time Warping

**DOI:** 10.3390/e21060593

**Published:** 2019-06-14

**Authors:** Guiji Tang, Bin Pang, Yuling He, Tian Tian

**Affiliations:** Department of Mechanical Engineering, North China Electric Power University, Baoding 071000, China

**Keywords:** hierarchical instantaneous energy density dispersion entropy, dynamic time warping, hierarchical dispersion entropy, gearbox, fault diagnosis

## Abstract

The accurate fault diagnosis of gearboxes is of great significance for ensuring safe and efficient operation of rotating machinery. This paper develops a novel fault diagnosis method based on hierarchical instantaneous energy density dispersion entropy (HIEDDE) and dynamic time warping (DTW). Specifically, the instantaneous energy density (IED) analysis based on singular spectrum decomposition (SSD) and Hilbert transform (HT) is first applied to the vibration signal of gearbox to acquire the IED signal, which is designed to reinforce the fault feature of the signal. Then, the hierarchical dispersion entropy (HDE) algorithm developed in this paper is used to quantify the complexity of the IED signal to obtain the HIEDDE as fault features. Finally, the DTW algorithm is employed to recognize the fault types automatically. The validity of the two parts that make up the HIEDDE algorithm, i.e., the IED analysis for fault features enhancement and the HDE algorithm for quantifying the information of signals, is numerically verified. The proposed method recognizes the fault patterns of the experimental data of gearbox accurately and exhibits advantages over the existing methods such as multi-scale dispersion entropy (MDE) and refined composite MDE (RCMDE).

## 1. Introduction

As an important component for transmitting energy and adjusting speed, gearbox is used in a wide variety of industrial machinery, such as wind turbines, aircraft engines, and automobiles [1,2]. Its failure is one of the main factors that cause the machinery to stop [3]. Thus, the accurate fault diagnosis of gearbox is highly required.

Generally, the existing fault diagnosis approaches can be classified into two types: Model-based and data-driven [4,5,6]. The model-based approach depends on an expansive system and dynamic knowledge [6]. The data-driven approach does not require an exhaustive system or dynamic knowledge, which is more conducive to intelligent diagnosis. Thus, the data-driven approaches have been widely used in gearbox fault diagnosis. Fault feature extraction is a key step in the data driven fault diagnosis methods and has a decisive impact on the final result [7]. At present, the typical and mature fault feature extraction algorithms are mainly based on processing the measurements such as vibration signal [8,9,10], acoustic signal [11,12], and temperature [13]. Among them, the algorithms based on vibration signal processing are favored by researchers because of their obvious advantages, such as low sensor price, simple signal acquisition process, and rich information contained in the signal. The complicated transmission path and running environment contribute to the nonlinear–nonstationary property and noise interference of the gearbox vibration signal, which increases the difficulty of fault feature extraction [14]. Hence, the conventional spectra analysis algorithms based on Fourier transform (FT) is not enough to accurately diagnose gearbox faults [15]. 

Based on the excellent characteristics of time-frequency analysis (TFA) in processing nonlinear–nonstationary signals, scholars have been committed to the development and introduction of various TFA algorithms to fault feature extraction of gearbox in the past two decades. Classical TFA algorithms include wavelet transform (WT) [16], windowed FT (WFT) [17], and Hilbert-Huang transform (HHT) [18]. Among them, WT and WFT are less adaptive. HHT is exploited by integrating an adaptive signal separation method named empirical mode decomposition (EMD) and Hilbert transform (HT). Compared with WT and WFT, HHT is more adaptive. Moreover, the TFA results of WT and WFT contain the information on the interference components, while HHT can exclude the unwanted information of the interference signals by EMD. The signal separation of EMD is based on the local extreme points fitting, which causes modal aliasing when dealing with signals with anomalous events such as noise, discontinuities, and shocks [19]. To improve the immunity of EMD to anomalous events, some noise-assisted forms of EMD, such as ensemble EMD (EEMD) [20] and complementary EEMD (CEEMD) [21], have been invented and present advantages over EMD in fault feature extraction of gearbox. However, how to adaptively select the added noise is an obstacle of EEMD and CEEMD. Some scholars tried to modify EMD by improving the extreme point fitting [22]. Moreover, some variants of EMD, such as intrinsic time-scale decomposition (ITD) [23], local mean decomposition (LMD) [24], and local oscillatory-characteristic decomposition (LOD) [25] were developed by modifying the envelope definition. However, they are also sensitive to noise. Some recently proposed signal decomposition algorithms such as variational mode decomposition (VMD) [26], morphological component analysis (MCA) [27], and empirical wavelet transform (EWT) [28] make great breakthroughs in overcoming the modal aliasing problem compared with those signal separation algorithms based on extreme point fitting. However, they are highly sensitive to parameter selection and are not conducive to online analysis. Considering the limitations of the previous signal separation algorithms, Bonizzi [29] put forward the singular spectrum decomposition (SSD) approach. SSD inherits the signal separation function of singular spectrum analysis (SSA) and does not need to manually select parameters. The effectiveness of SSD for processing vibration signals of rotating machinery and its superiority over the previous signal separation algorithms has been fully illustrated in previous studies [30,31,32]. Thus, it is combined HT for TFA in this work. In the introduction of the principle of HHT, Huang also gives the concept of instantaneous energy density (IED). The IED analysis has shown its advantages for enhancing impact fault features [33,34]. In this paper, a novel IED analysis method based on SSD and HT is proposed to inhibit the useless interferences. 

With the concept of data fusion, we expect to use reliable fault feature extraction methods and intelligent classifiers to realize intelligent diagnosis [35]. Complexity and regularity reflect the characteristic information of the signal [36]. Dispersion entropy (DE) is a novel proposed entropy indicator, which is able to quantify the complexity and regularity of the signal [37]. It makes some improvements compared with the previous entropy indicators such as approximate entropy (AE) and permutation entropy (PE). For example, DE is more efficient than AE. The information relates to the amplitude of the signal, which is considered in DE, while it is discarded by PE [38]. To better measure the information of the complex vibration signal of rotating machinery, some multi-scale forms of DE, such as multi-scale DE (MDE) [39] and refined composite MDE (RCMDE) [40], are proposed by introducing the coarse-grained and refined composite coarse-grained procedures. However, the coarse-grained and refined composite coarse-grained procedures can only detect the low-frequency information of the signal [41]. Thus, the hierarchical decomposition [42,43], which can probe the low-frequency and high-frequency information of the signal is combined with DE to develop the hierarchical DE (HDE) algorithm. Based on the merits of the IED analysis and HDE, we put forward the hierarchical instantaneous energy density dispersion entropy (HIEDDE) to better extract the fault information of gearbox. After the process of fault feature extraction, dynamic time warping (DTW) [44] is adopted to recognize the fault patterns. Compared with the other classifiers, fewer samples are required to form the template signal [45]. A novel gearbox fault diagnosis framework based on HIEDDE and DTW is formulated. Finally, the proposed fault diagnosis framework is tested by the experimental data of gearbox.

The outline of this study is as follows. In Section 2, the details of the proposed HIEDDE algorithm based on the IED analysis and the HDE algorithm are introduced. Section 3 briefly illustrates the principle of DTW. Section 4 introduces the specific implementation procedures of the proposed method. Section 5 presents the experimental test. Finally, a few conclusions are presented in Section 6.

## 2. Hierarchical Instantaneous Energy Density Dispersion Entropy

### 2.1. IED Analysis

The vibration signal of gearbox is usually accompanied by interferences. Thus, a novel IED analysis algorithm based on SSD and HT is introduced to reinforce the fault feature signal. The SSD algorithm is used to obtain the useful potential components. Then, HT is employed to detect the time-frequency information of these components. Finally, the IED signal is obtained based on the time-frequency information. 

#### 2.1.1. SSD

SSD is a parameter-less signal-processing algorithm that can reach the signal decomposition adaptively. It is regarded as a modified version of SSA as it achieves the adaptive selection of the embedding dimension and can complete the automatic principal components grouping. The SSD decomposition for a composite signal is mainly composed of four steps as follows [29].

**Step 1**: Trajectory matrix construction.

For a discrete signal *a*(*n*) that has *N* samples, *a*(*n*) = (*a*_1_, *a*_2_,…, *a_N_*), the form of its trajectory matrix ***A*** is closely related to the selection of embedding dimension. If the embedding dimension is selected as *K*, ***A*** will be a *K* × *N* matrix, whose *k*-th row is ***a****_k_* = (*a_k_*,…, *a_N_*, *a*_1_,…, *a_k_*_−1_). That is, $A={\left[a_{1}^{T},a_{2}^{T},\cdots ,a_{K}^{T}\right]}^{T}$.

How to choose the embedding dimension reasonably is a difficult problem of the original SSA algorithm. The following procedures are adopted in SSD to adaptively choose the embedding dimension at iteration *j*:

First, perform the calculation of the power spectral density (PSD) of the residual component *v_j_*(*n*) for iteration *j*, $v_{j}(n)=a(n)-\sum_{k=1}^{j-1}v_{k}(n)$ (*v*_0_(*n*)=*a*(*n*)). Then, the frequency *f*_max_ at the maximum peak of the calculated PSD is distinguished. When *j* = 1, the residual component is regarded as a trend item if *f*_max_ is smaller than a given threshold, which is set as 0.01*F_s_* in this paper, (*F_s_* represents the sampling frequency). In this situation, *K* is recommended as *N*/3. In other situations, *K* = 1.2*F_s_*/*f*_max_. 

**Step 2**: Singular valued decomposition.

The trajectory matrix ***A*** obtained in **Step 1** carries the mixed information of all component signals. Thus, it is subjected to singular valued decomposition (SVD):

(1) $$\begin{array}{l} A=P\Sigma Q^{T}\\ =\left[p_{1},p_{2},\cdots ,p_{K}\right]\left[\begin{array}{ccccc} \alpha_{1} & 0 & 0 & 0 & 0\\ 0 & \alpha_{2} & 0 & 0 & 0\\ \vdots & \vdots & \ddots & \vdots & 0\\ 0 & 0 & 0 & \alpha_{K} & 0 \end{array}\right]\left[\begin{array}{c} q_{1}^{T}\\ q_{2}^{T}\\ \vdots \\ q_{N}^{T} \end{array}\right]\\ =\alpha_{1}p_{1}q_{1}^{T}+\alpha_{2}p_{2}q_{2}^{T}+\cdots +\alpha_{K}p_{K}q_{K}^{T}\\ =A_{1}+A_{2}+\cdots +A_{K} \end{array}$$

where ***P***∈***R****^K^*^×^*^K^*, ***∑***∈***R****^K^*^×^*^N^*, ***Q***∈***R****^N^*^×^*^N^*. {*α*_1_, *α*_2_, …, *α_K_*} are the singular values in matrix***∑***, ***A****_i_*= *α_i_**p**_i_**q**_i_* represents the *i*-th principal component of ***A***.

**Step 3**: Principal components grouping and reconstruction of the *j*-th SSC.

As represented in Equation (1), the trajectory matrix is divided into *K* principal components by SVD. The key to the successful separation of a potential component is to sort these principal components reasonably. The following criterion is used in SSD for selecting principal components to reconstruct the SSC at the *j*-th iteration:

For *j* = 1, if a trend item is identified, only the first principal component ***A***_1_ is chosen as the useful principal component, and the first SSC is retrieved through the diagonal averaging of ***A***_1_.

Otherwise, and for *j* > 1, a subset principal components, whose left eigenvectors show a primary frequency in [*f*_max_ −*f*_1_, *f*_max_ + *f*_1_] (where *f*_1_ is estimated through the Gaussian interpolation of the PSD of *v_j_*(*n*)), are combined to form a matrix ***A***’. The *j*-th SSC is retrieved through the diagonal averaging of ***A***’.

**Step 4**: Setting stopping criterion.

Once a component SSC*_j_*(*n*) is retrieved, a corresponding residual signal can be obtained as:

(2) $$v_{j+1}(n)=v_{j}(n)-SSC_{j}(n).$$

*v_j_*_+ 1_(*n*) is then employed as the input to retrieve the (*j* + 1)-th SSC. If the result of the equation below is smaller than a given threshold, the iteration will be stopped.

(3) $$NMSE_{j}=\frac{\sum_{i=1}^{N}{\left(v_{j+1}(i)\right)}^{2}}{\sum_{i=1}^{N}{\left(x(i)\right)}^{2}}.$$

#### 2.1.2. IED Calculation Based on SSD and HT

After the SSD process, the composite signal *a*(*t*) can be expressed as the sum of *k* SSCs and a residual signal:

(4) $$a\left(t\right)=\overset{k}{\underset{j=1}{\sum }}SSC_{j}\left(t\right)+r\left(t\right),$$

where *SSC_j_*(*t*) represents the *j*-th SSC and *r*(*t*) is the residual signal.

Previous studies have indicated that SSD has stronger signal decomposition capability than the traditional signal decomposition approaches such as EMD and EEMD [30,31,32]. Thus, it is integrated with HT to make the time-frequency analysis. HT is used to estimate the instantaneous amplitude (IA) and instantaneous frequency (IF) of the decomposed components by referring to Reference [18]. The SSD-HT spectrogram can be constructed by lumping the IAs and IFs of all SSCs:

(5) $$SSD-HT\left(\omega ,t\right)=\overset{k}{\underset{j=1}{\sum }}a_{j}\left(t\right)\exp(i\int \omega_{j}\left(t\right)).$$

where *k* represents the number of the decomposed components, *a_j_*(*t*) and *ω_j_*(*t*) are the IA and IF of the *j*-th SSC, respectively.

We can further obtain the IED signal with the following form:

(6) $$IED\left(t\right)=\underset{\omega }{\int }{\left|SSD-HT\left(\omega ,t\right)\right|}^{2}d\omega .$$

The IED reflects the energy fluctuation of the signal over time.

However, some of the decomposed SSCs may be false components because the vibration signal of gearbox contains interferences. In this paper, the correlation coefficient between the original signal *a*(*t*) and the decomposed components is used to discard the false components [46]:

(7) $$\rho_{j}=\frac{COV\left(SSC_{j}\left(t\right),a\left(t\right)\right)}{\sigma_{j}\sigma_{a}},$$

where *ρ_j_* is the correlation coefficient between *a*(*t*) and the *j*-th SSC, *COV*(*SSC_j_*(*t*), *a*(*t*)) represents the covariance of *SSC_j_*(*t*) and *a*(*t*), and *σ_j_* and *σ_a_*, respectively, correspond to the variance of *SSC_j_*(*t*) and *a*(*t*).

If *ρ_j_* is larger than a threshold (which is set to 0.2 max (*ρ*_1_, *ρ*_1_,…, *ρ_k_*) in this paper), *SSC_j_*(*t*) is seen as a real component. All the real SSCs are selected out to calculate the IED to enhance the fault signatures of gearbox.

#### 2.1.3. Verification of the Fault Feature Enhancement Capability of IED

A mixed signal containing the distributed fault signal of gear and noise is tested to examine the fault feature enhancement capability of the IED analysis. The fault signal is generated using the model proposed by McFadden [47]:

(8) $$s_{\mathrm{faulty}}\left(t\right)=\overset{L}{\underset{k=0}{\sum }}S_{k}\left(1+a_{k}\left(t\right)\right)\cos(2\pi kzf_{r}t+\varphi_{k}+b_{k}\left(t\right)),$$

where *L* is the harmonic orders, *S_k_* and *φ_k_* are the amplitude and phase of the *k*-th meshing harmonic, *z* and *f_r_* represent the number of teeth and rotating frequency of the faulty gear, *a_k_*(*t*) is the amplitude modulation (AM) item and *b_k_*(*t*) represents the phase modulation (PM) item. The AM and PM items are, respectively, defined as:

(9) $$a_{k}\left(t\right)=\overset{M}{\underset{m=0}{\sum }}A_{km}\cos(2\pi mf_{r}t+a_{km}),$$

(10) $$b_{k}\left(t\right)=\overset{M}{\underset{m=0}{\sum }}B_{km}\cos(2\pi mf_{r}t+b_{km}),$$

where *M* is the total orders of the *k*-th meshing harmonic, *A_km_* and *a_km_* denote the amplitude and phase of AM, *B_km_* and *b_km_* represent the amplitude and phase of PM.

The other parameters are adopted as: *L* = 3, *S_k_* = 1, *z* = 30, *f_r_* = 12 Hz, *φ_k_* = 0, *A_km_* = 0.9, *a_km_* = 0, *B_km_* = 0.1, *b_km_* = 0. The meshing frequency can be calculated as: *f_m_* = 30 × 12 = 360 Hz. In order to meet the requirement of frequency spectrum analysis and sampling theorem, the sampling frequency should be at least 3–5 times the meshing frequency; we select the sampling frequency as 10,000 Hz. Moreover, the signal length is set as 8192 to ensure a higher accuracy of Fourier transform.

Equation (11) is adopted to produce the noise:

(11) n(t)=0.2randn(1,N),

where *N* is the signal length.

**Figure 1:**
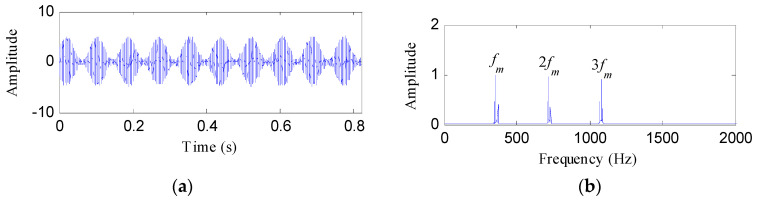
(**a**) The tested signal and (**b**) its amplitude spectrum.

By adding the noise to the fault signal, the tested signal is yielded as shown in Figure 1a. From its amplitude spectrum as represented in Figure 1b, there are three harmonics of meshing frequency. However, the rotating frequency of the faulty gear cannot be located.

**Figure 2:**
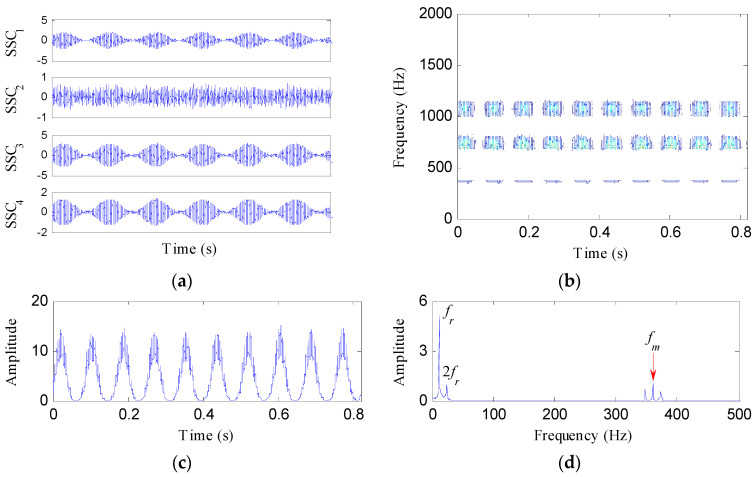
The IED analysis of the tested signal based on SSD and HT: (**a**) the decomposition results of SSD; (**b**) the SSD-HT spectrogram; (**c**) the IED signal; and (**d**) its amplitude spectrum.

Then, SSD is used to decompose the tested signal, and four SSCs are obtained as shown in Figure 2a. Table 1 shows the correlation coefficients between the tested signal and the SSCs. Based on the selection criterion as introduced in Section 2.2, SSC_2_ is determined as a false signal. The other three SSCs are demodulated by HT to obtain the SSD-HT spectrogram, as shown in Figure 2b. Obviously, the SSD-HT spectrogram reflects three frequency components, i.e., *f_m_*, 2*f_m_* and 3*f_m_*. Figure 2c represents the IED signal. From its amplitude spectrum as shown in Figure 2d, the first and second harmonics of the rotating frequency of the faulty gear and the meshing frequency are clearly located. The fault feature enhancement capability of the IED analysis is verified.

**Figure 3:**
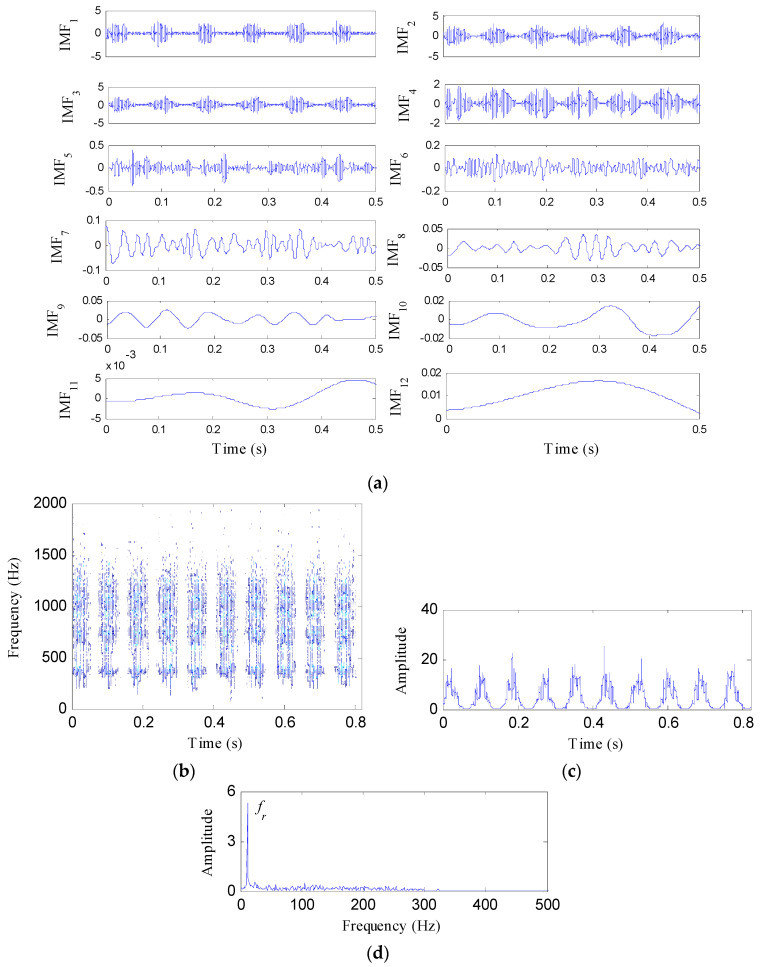
The IED analysis of the tested signal based on EMD and HT: (**a**) The decomposition results of EMD; (**b**) the EMD-HT spectrogram; (**c**) the IED signal; and (**d**) its amplitude spectrum.

For comparison, the simulated gear fault signal is also analyzed by the IED analysis based on EMD and HT. Twelve components are generated after the EMD process, as shown in Figure 3a. From the waveforms of these components, it can be seen that the fifth to twelfth components are false components. Thus, the first four components retrieved by EMD are analyzed by HT to acquire the EMD-HT spectrogram, as depicted in Figure 3b. Figure 3c,d represent the corresponding IED signal and its amplitude spectrum, respectively. Only the rotating frequency of the faulty gear appears in Figure 3d, while its harmonics and the meshing frequency are invisible. The superiority of the IED analysis based on SSD is demonstrated.

### 2.2. Hierarchical Dispersion Entropy

#### 2.2.1. Dispersion Entropy

DE is a novel entropy index for quantifying the certainty of signals. Its advantages over the traditional entropy methods have been investigated by many scholars [38,39,40]. For a signal that has *N* sampling points, *a*(*n*) = (*a*_1_, *a*_2_,…, *a_N_*), its DE can be calculated through the following procedures [37]:

(1) A new signal *b*(*n*) = (*b*_1_, *b*_2_, …, *b_N_*), with *b_i_* ∈ (0,1), is obtained by applying normal cumulative distribution functions (NCDF) to the original signal *a*(*n*):

(12) $$b_{i}=\frac{1}{\sigma \sqrt{2\pi }}\overset{a_{i}}{\underset{-\infty }{\int }}e^{\frac{-{\left(t-u\right)}^{2}}{2\sigma^{2}}}dt,$$

where *σ*^2^ and *u* indicate the variance and mean of *a*(*n*), respectively.

(2) *b*(*n*) is mapped to the integer range of [1, 2, …, *c*] with the following formula:

(13) $$Z_{i}^{c}=round\left(c\cdot b_{i}+0.5\right).$$

(3) An embedded vector $Z_{i}^{m,c}$ is formed with embedding dimension *m* and time delay *τ*:

(14) $$Z_{i}^{m,c}=\left\{Z_{i}^{c}{,Z}_{i+\tau }^{c},\ldots {,Z}_{i+\left(m-1\right)\tau }^{c}\right\},\;i=1,2,\ldots ,\left(m-1\right)\tau .$$

(4) The corresponding dispersion pattern $\pi_{\upsilon_{0},\upsilon_{1},\ldots ,\upsilon_{m-1}}$ is obtained with the following definition:

(15) $$Z_{i}^{c}=\upsilon_{0},\;Z_{i+\tau }^{c}=\upsilon_{1},\ldots ,\;Z_{i+\left(m-1\right)\tau }^{c}=\upsilon_{m-1}.$$

(5) The relative frequency of each dispersion pattern is computed as:

(16) $$P\left(\pi_{\upsilon_{0},\upsilon_{1},\ldots ,\upsilon_{m-1}}\right)=\frac{N_{a}\left(\pi_{\upsilon_{0},\upsilon_{1},\ldots ,\upsilon_{m-1}}\right)}{N-\left(m-1\right)\tau }.$$

(6) With referring to the definition of Shannon entropy, the DE of *a*(*n*) is computed as:

(17) $$DE\left(a,m,c,\tau \right)=-\overset{c^{m}}{\underset{\pi =1}{\sum }}P\left(\pi_{\upsilon_{0},\upsilon_{1},\ldots ,\upsilon_{m-1}}\right)\ln(P\left(\pi_{\upsilon_{0},\upsilon_{1},\ldots ,\upsilon_{m-1}}\right)).$$

Previous studies [36,37,38,39,40] recommend that the parameters selection for the DE algorithm should be: *m* = 2, *c* = 6, *τ* = 1. Thus, we take this suggestion in all the calculation of DE in this paper.

#### 2.2.2. Hierarchical Dispersion Entropy

In order to better detect the regularity of the complex signals, some multi-scale forms of DE, such as MDE and RCMDE, have been developed. However, previous studies reveal that the multi-scale analysis algorithms only consider the low-frequency components of the signal and cannot completely detect the hidden fault features [41,42,43]. Thus, a hierarchical DE (HDE) algorithm is proposed by integrating the hierarchical decomposition and DE to quantify the information of the signal more comprehensively.

For a 1 d signal ***A*** = (*a*_1_, *a*_2_,…, *a_N_*) with the length of *N* (*N* = 2*^n^*, *n* represents a positive integer), its calculation procedures of HDE include:

(1) An average operator *q*_0_ and a difference operator *q*_1_ are respectively constructed as follows:

(18) $$q_{0}\left(A\right)=\frac{a_{2j}+a_{2j+1}}{2},\;j=1,2,\ldots ,2^{n-1},$$

(19) $$q_{1}\left(A\right)=\frac{a_{2j}-a_{2j+1}}{2},\;j=1,2,\ldots ,2^{n-1},$$

where *q*_0_(***A***) and *q*_1_(***A***) carry the low-frequency and high-frequency features of ***A*** at scale 2, respectively.

(2) The matrix expression of the operators *q_j_* (*j* = 0 or 1) is obtained as the following form:

(20) $$q_{j}=\left[\begin{array}{ccccccc} \frac{1}{2} & \frac{{\left(-1\right)}^{j}}{2} & 0 & 0 & \ldots & 0 & 0\\ 0 & 0 & \frac{1}{2} & \frac{{\left(-1\right)}^{j}}{2} & \ldots & 0 & 0\\ 0 & 0 & 0 & 0 & \ldots & \frac{1}{2} & \frac{{\left(-1\right)}^{j}}{2} \end{array}\right].$$

(3) In order to perform the hierarchical analysis on the signal ***A***, the above operators have to be employed iteratively. Let *p* ∈ *N*, a vector [*β*_1_, *β*_2_,…, *β_p_*]∈{0, 1} can be designed to describe the integer *d*:

(21) $$d=\overset{p}{\underset{j=1}{\sum }}\beta_{j}2^{p-j}.$$

It can be inferred that for a given integer *E*, there is a unique vector [*β*_1_, *β*_2_,…, *β_p_*] corresponding to it.

(4) The hierarchical component ***A****_k_*,*_d_* (where *k* and *d* are the layer number and node number, respectively) is expressed as:

(22) $$A_{k,d}=q_{\beta_{1}}•q_{\beta_{2}}•\ldots •q_{\beta_{p}}•A.$$

(5) The DE of the hierarchical component at node *d* and layer *k* is calculated to obtain the HDE:

(23) $$HDE\left(A,k,d,m,c,\tau \right)=DE\left(A_{k,d},m,c,\tau \right).$$

**Figure 4:**
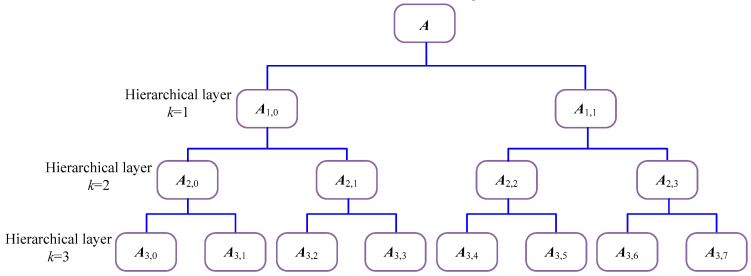
Hierarchical decomposition with three layers.

**Figure 5:**
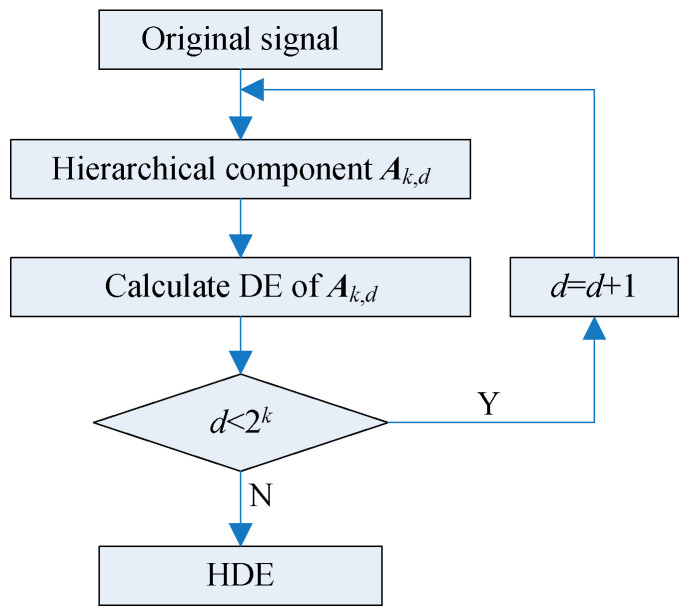
Flowchart of the HDE algorithm.

The hierarchical decomposition of signal ***A*** with three layers is displayed in Figure 4 to better illustrate its principle. It can be found that there are 2*^k^* hierarchical components at layer *k*. Figure 5 represents the calculation flowchart of HDE. In this paper, the HDE in layer 3 (which contains 8 nodes), which is used to characterize the information of the signal.

#### 2.2.3. Effectiveness Evalution of HDE

**Figure 6:**
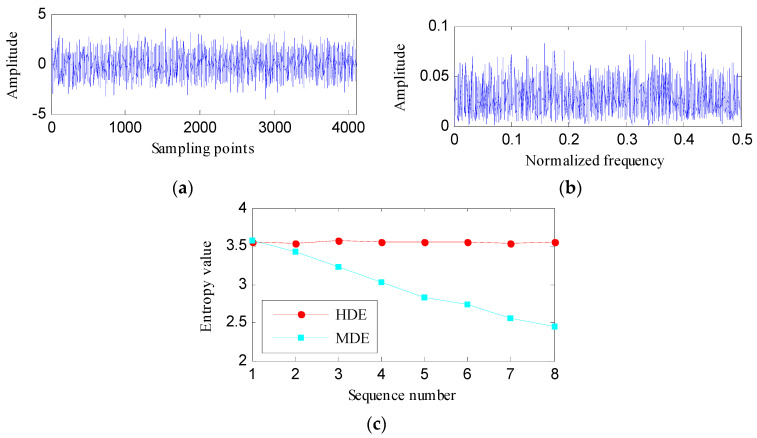
(**a**) The studied GWN signal; (**b**) its amplitude spectrum; and (**c**) its HDE and MDE.

A Gaussian white noise (GWN) with 4096 sampling points, as depicted in Figure 6a is studied to compare the effectiveness of HDE and MDE. From its magnitude spectrum, as shown in Figure 6b, we can deduce that the complexity of GWN in different frequency bands is almost the same. The HDE (with three layers) and MDE (with eight scales) of the tested GWN are computed, as displayed in Figure 6c. The HDE values of eight hierarchical components are almost equal. Whereas, the MDE value gradually decreases as the scale increases. In theory, the DE values that correspond to different frequency bands should not change much. The HDE more realistically reflects the complexity of the studied GWN signal compared to the MDE.

**Figure 7:**
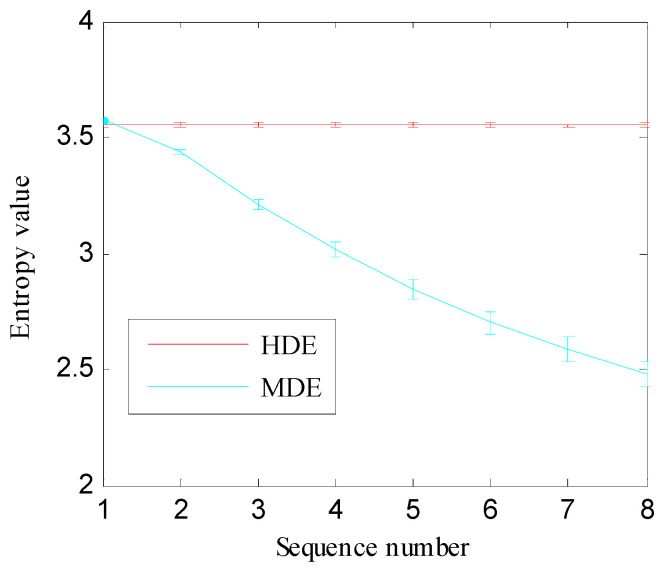
The error bars of HDE and MDE.

In addition, 100 independent sets of GWN (each set comprises 4096 samples) are tested to reveal the advantage of HDE over MDE. The median and standard deviation (SD) of HDE and MDE are calculated to obtain the error bars as shown in Figure 7. The SD of HDE in different nodes is very tiny, which illustrates the HDE algorithm has strong stability. However, the SD of MDE increases monotonically with scale. Starting from sequence number 2, the SD value of MDE is significantly larger than the SD value of HDE, which implies that the HDE algorithm is more stable.

### 2.3. Hierarchical Instantaneous Energy Density Dispersion Entropy

**Figure 8:**
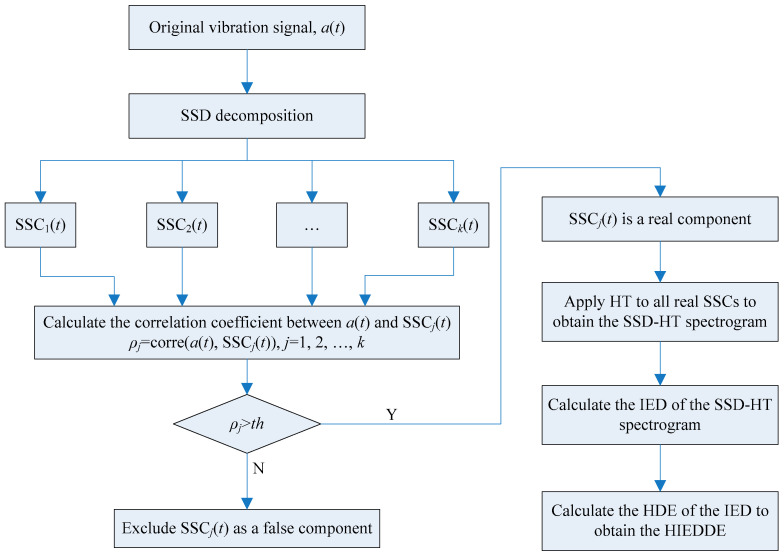
Flowchart of the HIEDDE algorithm.

Integrating the merits of IED and HDE, a novel fault signatures algorithm named hierarchical instantaneous energy density dispersion entropy (HIEDDE) is put forward. In this algorithm, the IED analysis is first used for fault features enhancement. The HDE process is then employed to quantify the information of the IED signal to get the HIEDDE. Figure 8 shows the flowchart of the HIEDDE algorithm. Total five procedures are needed to implement the HIEDDE algorithm:

(1) Apply the SSD algorithm to the vibration signal to generate *k* SSCs.

(2) Calculate the correlation coefficient between the original signal and the *j*-th SSC, *ρ_j_* (*j* = 1, 2,…, *k*). If *ρ_j_* is larger than a threshold, as given in Section 2.2, SSC*_j_*(*t*) is seen as a real component. Otherwise, SSC*_j_*(*t*) is excluded as a false component.

(3) Apply HT to all real SSCs identified in step (2) to obtain the SSD-HT spectrogram.

(4) Calculate the IED signal of the SSD-HT spectrogram.

(5) Calculate the HDE of the IED signal to obtain the proposed HIEDDE.

## 3. Dynamic Time Warping

DTW was originally developed for speech classification [44]. In recent years, it has been introduced to the field of mechanical fault diagnosis [45]. The advantage of DTW over existing classifiers is that it requires only a small number of samples to create the template signal while ensuring high classification accuracy. It is used to qualify the similarity between two time sequences based on the DTW distance, which is calculated by finding the optimal alignment. The smaller DTW distance between the two signals represents the higher similarity. The following gives the specific description of DTW:

Given two time series *P* = {*p_i_*}, *I* = 1, …, *m* and *Q* = {*q_i_*}, *I* = 1, …, *n*, where *m* and *n* represent the length of *P* and *Q*, respectively. A *m*×*n* distance matrix ***D*** is firstly constructed with the elements *d*(*p_i_*, *q_j_*), which are the Euclidean distance between the points *p_i_* and *q_j_*. Then, a path through the matrix ***D****,* which minimizes the cumulative distance between the two sequences can be found. DTW distance corresponds to the path with minimal warping cost:

(24) $$DTW\left(P,Q\right)=\min\sqrt{\sum_{k=1}^{K}w_{k}},$$

where *w_k_* is the matrix element and also belongs to the *k*-th element of a warping path *W*. The warping path needs to reach the following three constraints:

(a) Boundary condition: the starting point is *w*_1_ = (1,1) and the ending point is *w_K_* = (*m*, *n*).

(b) Continuity: if *w_k_* = (*a*, *b*) and *w*_*k*-1_ = (*a*’, *b*’), then (*a* − *a*’) ≤ 1 and (*b* − *b*’) ≤ 1.

(c) Monotonicity: if *w_k_* = (*a*, *b*) and *w*_*k*-1_ = (*a*’, *b*’), then (*a* − *a*’) ≥ 0 and (*b* − *b*’) ≥ 0.

The Warping path can be determined by using dynamic programming as follows:

(25) $$\gamma \left(i,j\right)=d\left(p_{i},q_{j}\right)+\min\{\begin{array}{c} \gamma \left(i-1,j-1\right)\\ \gamma \left(i-1,j\right)\\ \gamma \left(i,j-1\right) \end{array},$$

where *γ*(*i*, *j*) is the sum of *d*(*p_i_*, *q_j_*) and the minimum cumulative distance of three adjacent elements.

## 4. The Proposed Fault Diagnosis Method

**Figure 9:**
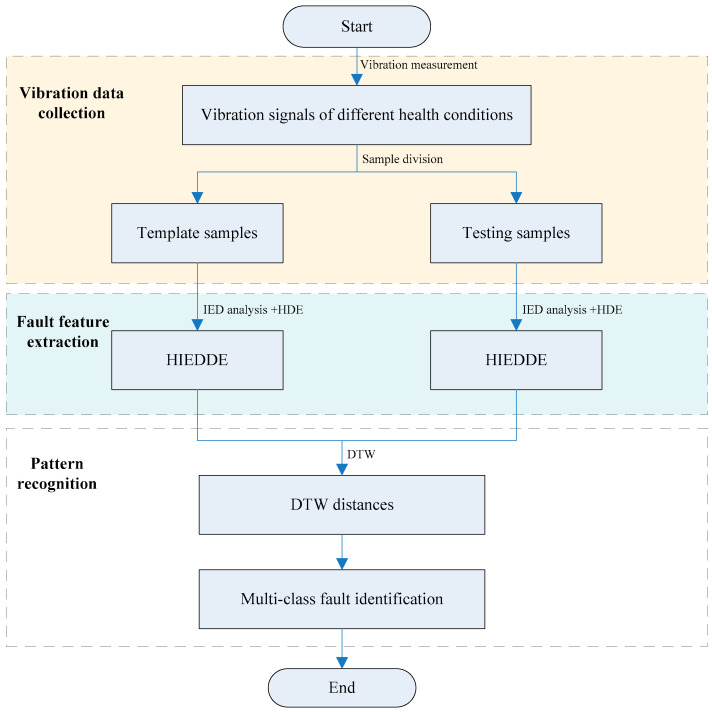
Implementation process of the proposed method.

A novel fault diagnosis method of gearbox based on the HIEDDE and DTW is proposed in this study. Figure 9 represents its implementation process, which is mainly composed of three steps:

(1) Collect the vibration data of gearbox under different health conditions. The vibration data of each condition is divided into some non-overlapping data groups. For each state, one data group is used for template sample and the other groups are used as testing samples. The number of the template samples is equal to the number of fault types.

(2) Calculate the HIEDDE of the vibration data as the fault features. The hierarchical layer is set to three so that the HIEDDE of each data sample contains the information of eight hierarchical components. 

(3) Employ DTW for fault pattern recognition. Based on the HIEDDE fault features, the DTW distance between the testing samples and template samples are calculated, and the fault type of the testing sample can be recognized based on the label of the template sample with the smallest distance.

## 5. Experimental Analysis

**Figure 10:**
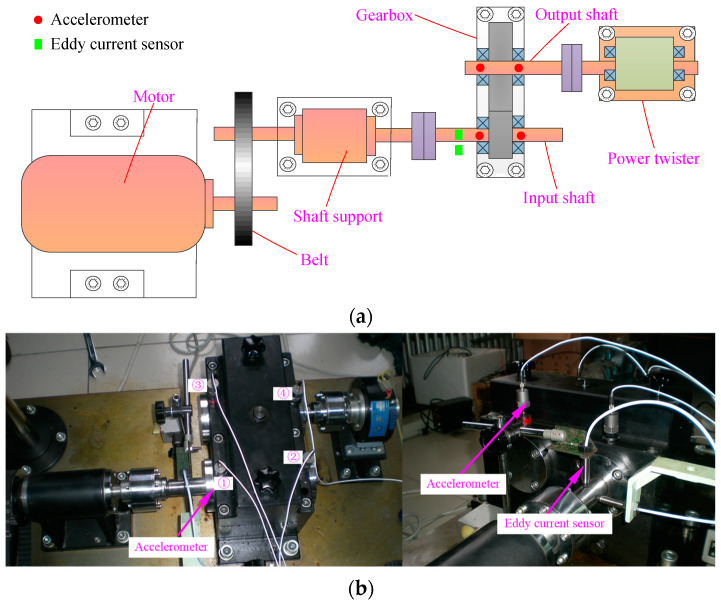
(**a**) The structure sketch of the test stand; (**b**) the sensors placement.

In this section, the presented fault diagnosis approach is employed to analyze the experimental data of gearbox under different states to test its validity. The data is obtained through the QPZZ-II rotating machinery fault simulation experiment platform. From the schematic diagram of the experiment platform, as depicted in Figure 10a, it can be found that the platform for gearbox faults simulation mainly consist of electric motor, belt drive, shaft support, gearbox, and power twister. The gearbox contains a small gear with 55 teeth installed on the input shaft and a big gear with 75 teeth mounted on the output gear. Table 2 represents the parameters illustration of the two gears. Four accelerometers were mounted on the housing of gearbox to measure the vertical vibration and two eddy current sensors are responsible for monitoring the radial vibration of the input bearing. The installation positions of the sensors are marked in Figure 10a. Figure 10b shows the sensor distribution from two perspectives. As displayed, the four accelerometers are numbered ①~④, respectively. The rotating speed of the input shaft in the experiment is 880 r/min. The meshing frequency of the small gear and big gear is 807 Hz. The sampling frequency is 5120 Hz. 

**Figure 11:**
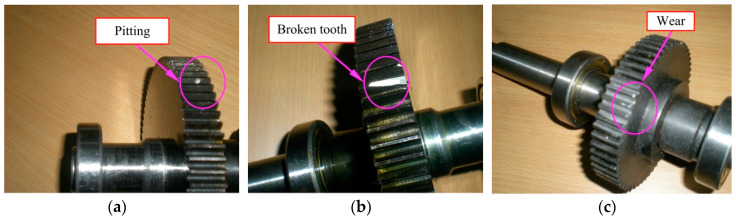
Gear damage faults: (**a**) pitting fault; (**b**) broken tooth fault; and (**c**) wear fault.

Experimental signals were collected under four health conditions including: (1) Normal condition; (2) a pitting tooth (PT) on the output gear; (3) a broken tooth (BT) on the output gear; (4) some wear teeth (WT) on the input gear; and (5) a compound fault condition with a BT on the output gear and a WT on the input gear (BT-WT). Figure 11 represents the damaged gears with different fault types employed in the test. For each state, the experimental signal is segmented into 41 data groups with the length of 4096. In order to ensure the independence of each group and test the effectiveness of the proposed method more reasonably and strictly, the data groups are non-overlapping. One data group of each state is selected as the template sample and the other 40 groups are regarded as the testing samples. Table 3 represents the details of the experimental signals. 

**Figure 12:**
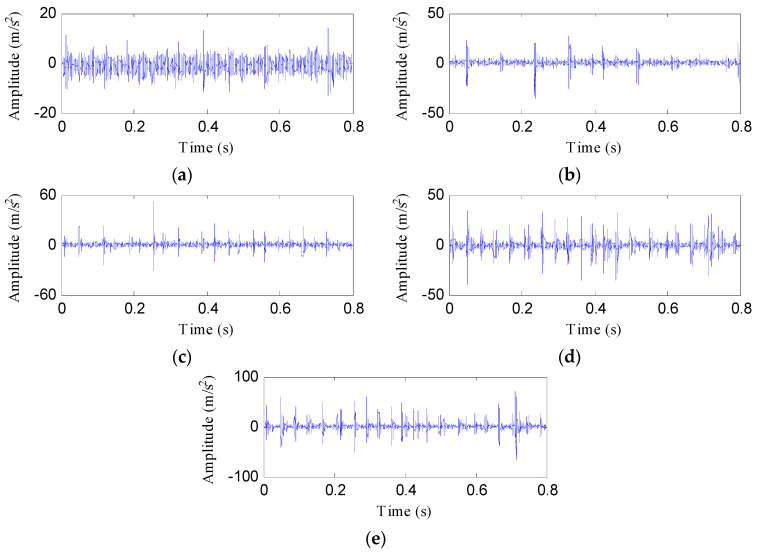
Data samples of the four health conditions: (**a**) Normal; (**b**) PT fault; (**c**) BT fault; (**d**) WT fault; and (**e**) BT-WT.

One testing sample of each health condition is used to illustrate the process of the proposed fault diagnosis approach. Figure 12 shows these samples under different health conditions. Despite the impacts of the data samples under the four fault states (PT, BT, WT, and BT-WT) being more prominent than the normal state, the fault states cannot be recognized because their impact features are somewhat similar.

**Figure 13:**
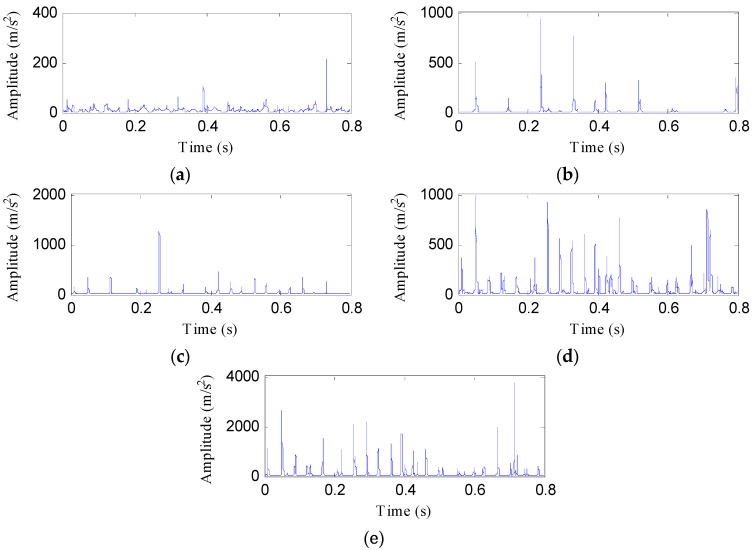
The IED signal of the four health conditions: (**a**) Normal; (**b**) PT fault; (**c**) BT fault; (**d**) WT fault; and (**e**) BT-WT.

**Figure 14:**
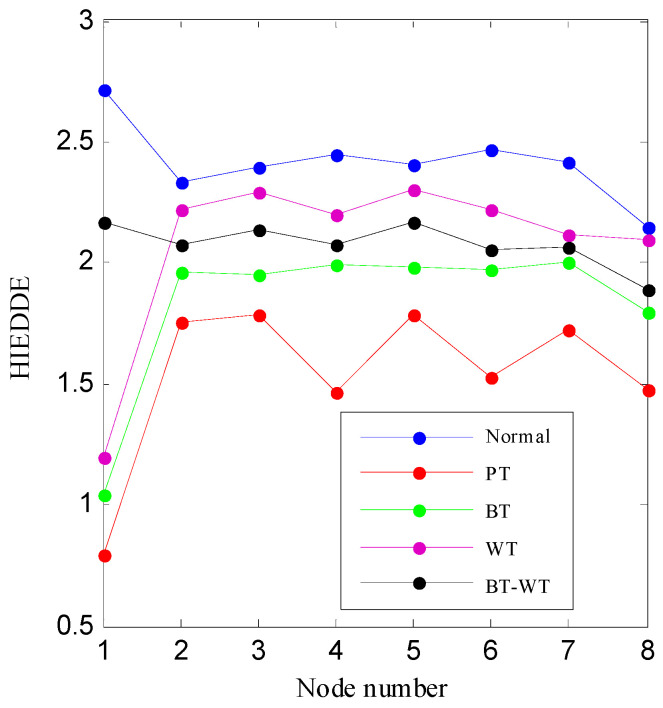
The HIEDDE fault features of the four data samples shown in Figure 11.

**Figure 15:**
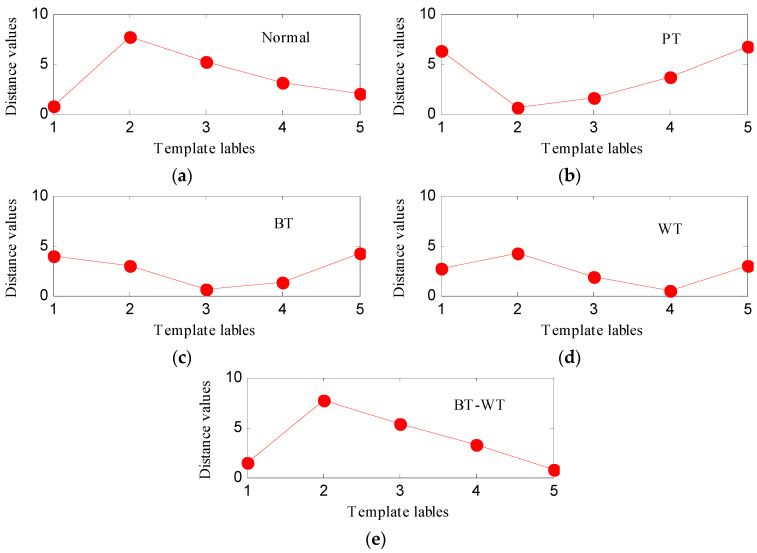
The similarity comparing results based on DTW: (**a**) Normal state; (**b**) PT; (**c**) BT; (**d**) WT; and (**e**) BT-WT.

**Figure 16:**
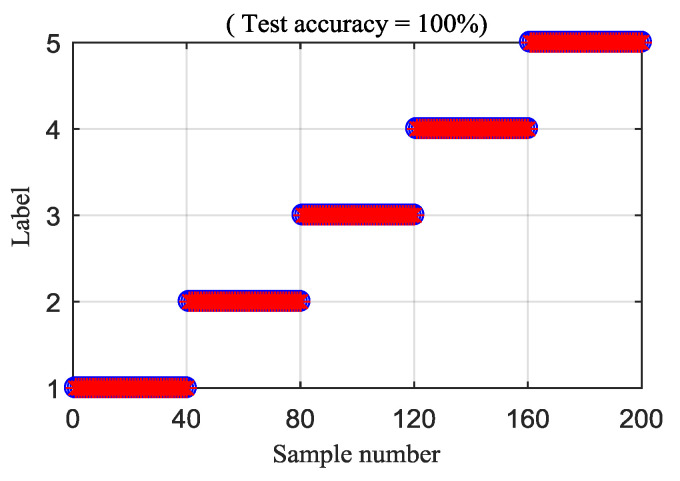
Classification rate of the proposed HIEDDE (IED-HDE) algorithm.

Then, the IED analysis is applied to the five selected data samples and the acquired corresponding IED signals are shown in Figure 13. Compared to the original signals, the impacts of the IED signal are more obvious as the noise is inhibited by the IED analysis. The HDE of the IED signals are calculated to obtain the HIEDDE fault features, as illustrated in Figure 14. The HIEDDE fault features under the four health conditions can be distinguished easily. The HIEDDE values of the eight nodes under health state are bigger than the three fault states. This implies that the signals under fault states are more regular. Based on the HIEDDE fault features, the DTW distances between the testing samples and the template samples are calculated and the results are represented in Figure 15. The labels of the five template samples correspond to the normal condition, PT fault, BT fault, WT and BT-WT faults are 1, 2, 3, 4, and 5, respectively. Figure 15a shows that the DTW distances between the testing sample under the normal condition and the five template samples have the minimum value at template label 1, which indicates that the health condition type of the normal state is accurately recognized. At the same time, we can find that the fault types of the testing samples under the four fault states are all identified based on the results shown in Figure 15b–e. Figure 16 shows the classification rate of the total 200 groups of testing data samples under the four health conditions by using the proposed method (HIEDDE-DTW). The classification rate is 100%. 

**Figure 17:**
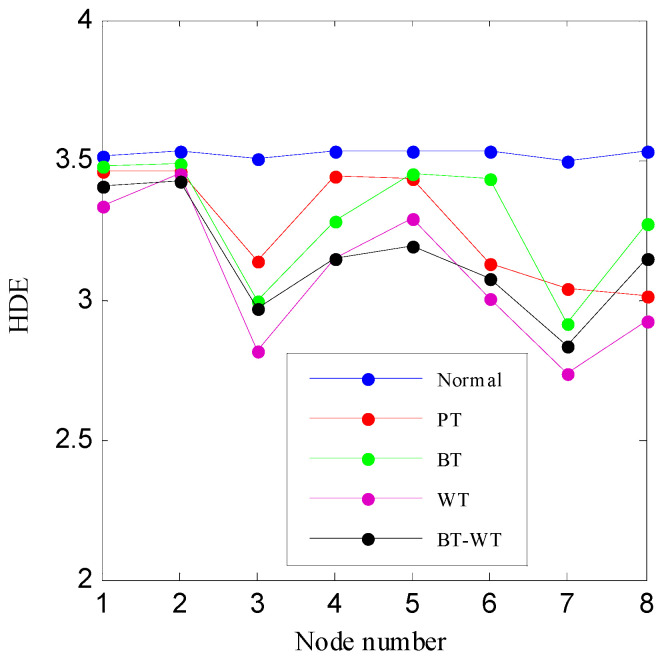
The HDE fault features of the four data samples shown in Figure 11.

**Figure 18:**
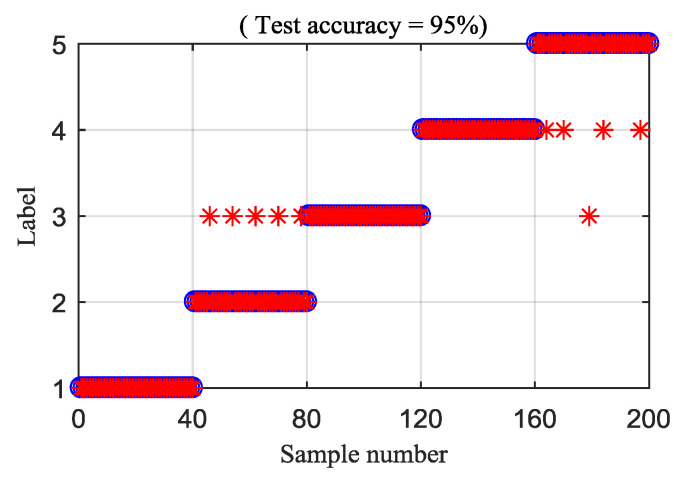
Classification rate of direct application of HDE.

HIEDDE is the combination of these two algorithms, i.e., the IED analysis and the proposed HDE algorithm. In order to illustrate the significance of the combination of the two algorithms, the HDE algorithm is directly applied to the five original data samples, as shown in Figure 12, and the corresponding HDE fault features are displayed in Figure 17. The HDE values of some nodes under different health conditions are almost coincidental. The difference of the HDE fault feature under the four health conditions shown in Figure 17 is not as high as that of the HIEDDE fault features under the five health conditions displayed in Figure 14, which illustrates that the IED analysis can help to enhance the fault features. Figure 18 shows the classification rate of all testing samples by using the HDE fault features. Five testing samples of the PT fault are judged as the BT fault, and five testing samples of the BT-WT fault are misclassified, which enables the classification rate to be 95%. 

**Figure 19:**
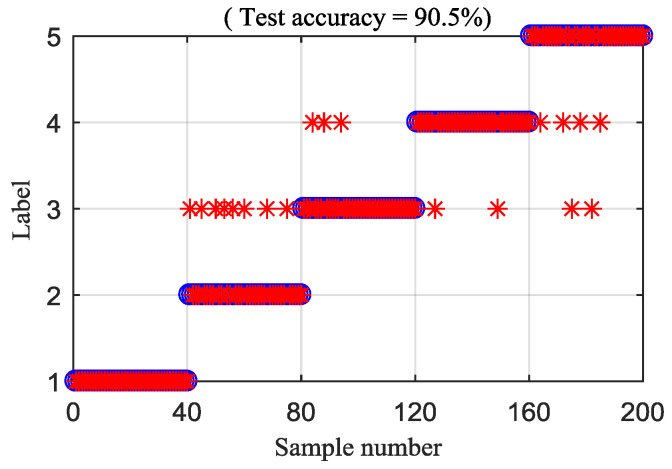
Classification rate of the IED-MDE method.

**Figure 20:**
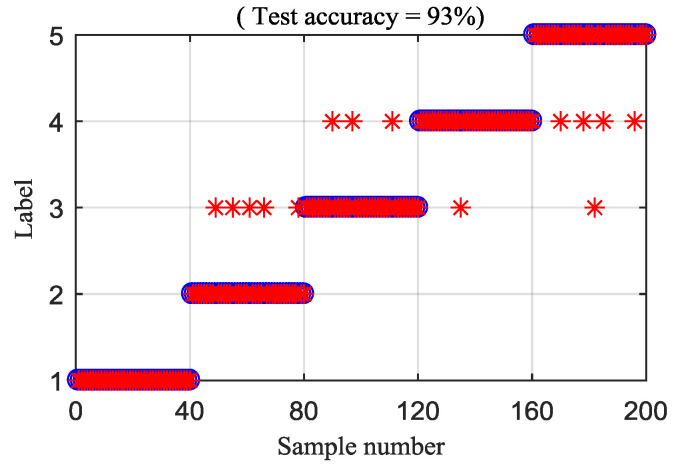
Classification rate of the IED-RCMDE method.

To explain the superiority of the proposed method, the MDE and RCMDE algorithms are integrated with the IED analysis to analyze the experimental signals. Figure 19 and Figure 20 illustrate the DTW classification results of the IED-MDE and IED-RCMDE approaches, respectively. The classification rate of the IED-MDE and IED-RCMDE approaches are 90.5% and 93%, respectively. The superiority of the proposed method is emphasized by the comparing results.

## 6. Conclusions

To make an accurate fault diagnosis of gearbox, a novel fault diagnosis framework is put forward by combing HIEDDE and DTW. The major innovations can be summarized as follows: (1) The IED analysis based on SSD and HT is introduced for fault feature enhancement of gearbox signals; (2) a novel entropy algorithm named HDE, which is able to quantify the information of the low-frequency and high-frequency components of signals, is presented; and (3) the advantages of IED and HDE are integrated to develop the novel fault feature extraction algorithm, i.e., HIEDDE. 

The analysis results of a simulated gear fault signal illustrate the fault feature enhancement ability of the IED analysis. The analysis results of the GWN signal demonstrate that the HDE algorithm is more stable and has higher accuracy compared with the MDE algorithm. The proposed method is used to identify the experimental fault patterns of gearbox. The results indicate the proposed method is able to recognize the fault patterns of gearbox accurately and has advantages over the existing methods.

It should be noticed that the validity of the proposed method is only studied by the experimental data obtained on the laboratory test bench. While satisfactory results have been obtained, the validity of the proposed method for practical engineering data needs to be further studied. The degree of fault difference between fault samples and the complexity of the fault may affect the accuracy of the proposed method. Moreover, the number of fault types that the proposed method can diagnose depends on the number of fault templates in the sample library. Only by establishing a rich sample library can the proposed method better solve the problem of gearbox fault diagnosis in engineering equipment.

## Figures and Tables

**Table 1:** The correlation coefficients between the tested signal and its singular spectrum components.

Decomposition Components	SSC_1_	SSC_2_	SSC_3_	SSC_4_
Correlation coefficients	0.4913	0.1084	0.7381	0.5731

**Table 2:** Parameters illustration of the two gears.

Gears	Modulus	Number of Teeth	Rotating Frequency	Materials
Small gear	2	55	14.67 Hz	S45C
Big gear	2	75	10.76 Hz	S45C

**Table 3:** Description of the experimental data.

Fault Type	Label	Number of Template Signal	Number of Testing Samples
Normal	1	1	40
PT	2	1	40
BT	3	1	40
WT	4	1	40
BT-WT	5	1	40
